# Supplementary material for: Impact of SchisandraChinensis Bee Pollen on Nonalcoholic Fatty Liver Disease and Gut Microbiota in HighFat Diet Induced Obese Mice
Source: Nutrients. 2019 Feb 6;11(2):346. doi: 10.3390/nu11020346 (PMC6412546; doi:10.3390/nu11020346)
Supplement: Supplementary file 1 [file nutrients-11-00346-s001.pdf]

Supporting information

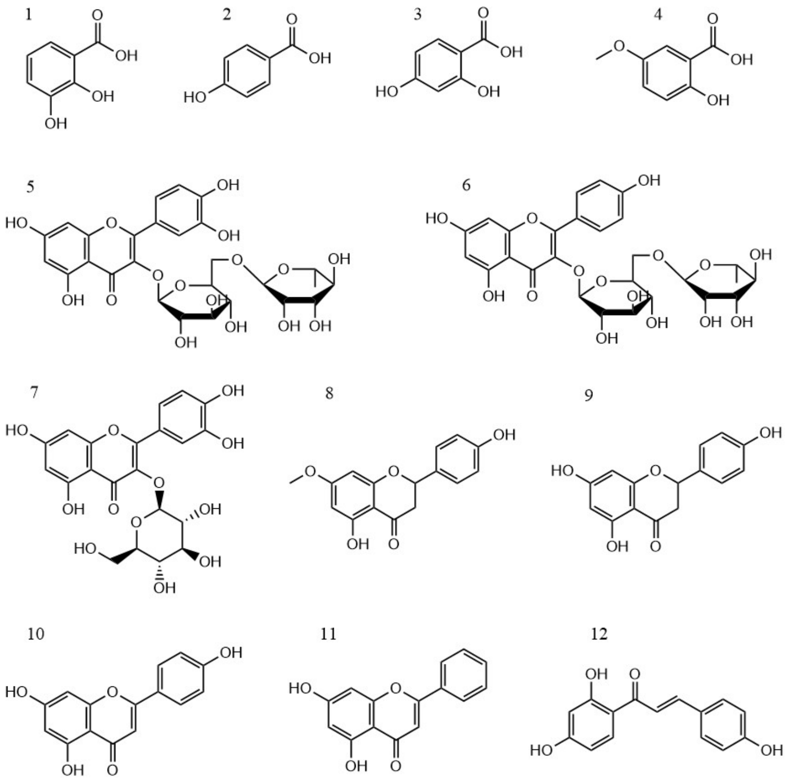

Figure 1. The structure of phenolic compounds identified in SCPE.

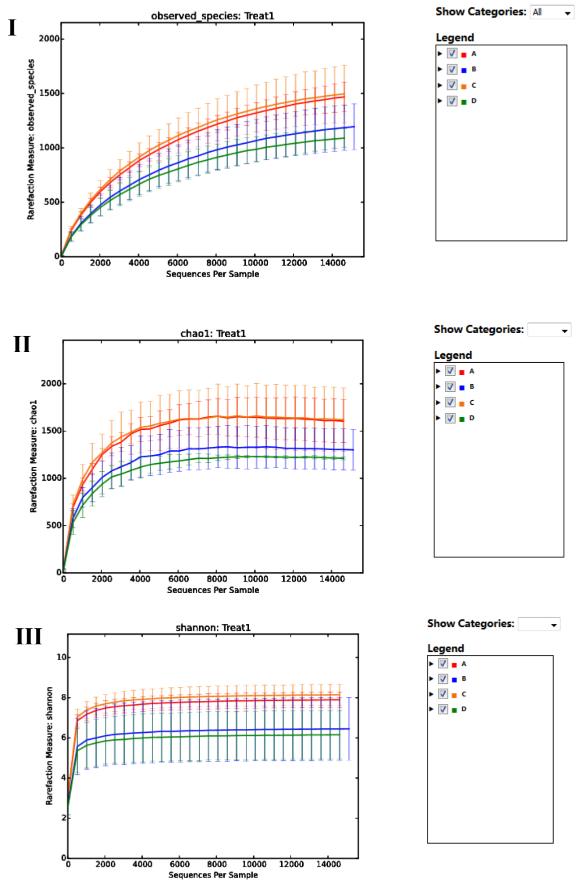

Fig.S2 Alpha diversity analysis of V3-V4 MiSeq sequencing reads of the 16SrRNA gene in different treated mice. I: Rarefaction curve; II: chao1 curve; III: Shannon curve. A: LFD (mice fed with low fat diet); B: HFD mice (mice fed with high fat diet); C: HFD+LE mice (mice fed with high fat diet and 7.86 g/kg BW of SCPE), D: HFD+HE mice (mice fed with high fat diet and 15.72 g/kg BW of SCPE)

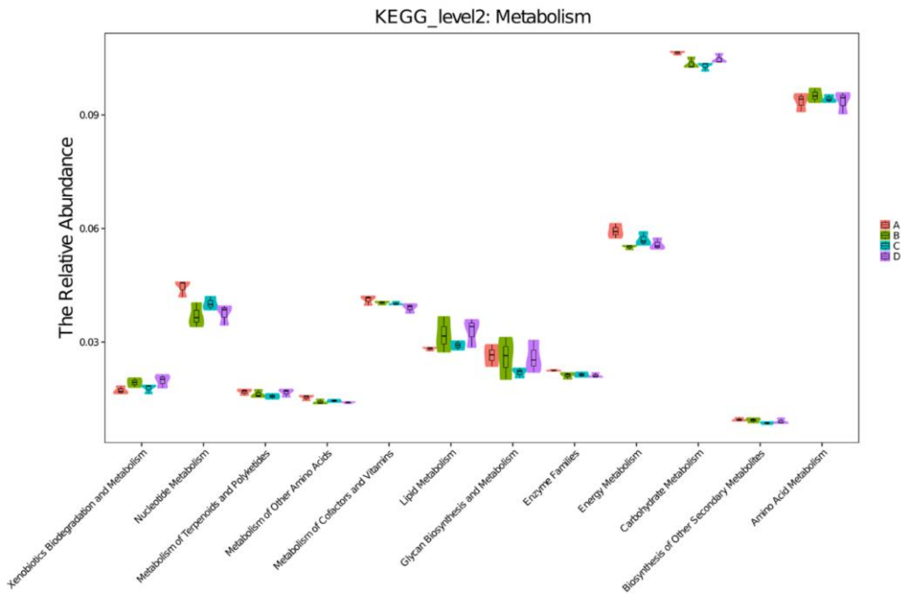

Fig. S3. Functional prediction of gut microbiota. A: LFD (mice fed with low fat diet); B: HFD mice (mice fed with high fat diet); C: HFD+LE mice (mice fed with high fat diet and 7.86 g/kg BW of SCPE), D: HFD+HE mice (mice fed with high fat diet and 15.72 g/kg BW of SCPE)
